# A Metropolitan Secretary's Optimism

**Published:** 1912-01-13

**Authors:** P. Michelli

**Affiliations:** Secretary. Dreadnought Hospital, Greenwich, S.E.


### A METROPOLITAN SECRETARY'S OPTIMISM.

Sir,—In answer to your letter:

(*a*) An estimate of the effect of the Insurance Act upon Voluntary Hospitals must be mainly guess-work and I am of opinion that it will probably not be so great as has been anticipated in some quarters. The speeches of Mr. Lloyd George and the agitation which has taken place in the country have both tended to make clear to the public that the hospitals will be at least as much in need of support as before.

(*b*) There appears no reason therefore why Voluntary Hospitals should not be able to maintain the same number of beds as in the past.

(*c*) I am of opinion that insured persons should not be excluded from hospital treatment. A case which is suitable on medical or surgical grounds for admission cannot obtain proper treatment outside hospital except in a nursing home, where the charges would be prohibitive. I do not think it would be wise to lay down any fixed rule for contributions from insured persons. There seems no reason why the approved societies under the Act should not contribute a voluntary sum to the hospitals on the basis of the number of their members treated, just as is done now by comparatively small bodies of organised workmen in such concerns as the South Metropolitan Gas Company.

(*d*) It seems doubtful whether Mr. Lloyd George has ever stated that insured persons would have no right to go to hospitals. In any case I do not think it would be possible to exclude them from relief on that ground. If such action were taken, the only alternative for them would be the Poor Law infirmaries, where there are I believe a number of empty beds.

Looking at the question as a whole I believe that voluntary hospitals have nothing to gain by extracting a fixed contribution for each insured person.* Such charges cannot, under the financial provisions in the Act, be sufficient to help their finances materially, even if they could be enforced, and they would be a serious encroachment on the principle of voluntary contributions which alone gives us complete control over our own hospitals.

The campaign in the public press, in which my fellow secretaries put forth every reason why the public should not subscribe, must be detrimental to our cause, and I believe that our best policy is quietly to continue our work in the confident hope that our appeals will be responded to by the charitable as generously as in the past, while we look to the working-man to support us as liberally through the medium of the approved society as he now does through his local sick or benefit club.

P. Michelli, Secretary.

Dreadnought Hospital, Greenwich, S.E.

* If Mr. Michelli will refer to The Hospital of July 29, 1911, page 451, he will see that Mr. Lloyd George told the deputation of Voluntary Hospital managers that they should say to the patients, "Are you insured? If they were, then they had no right to come to the hospitals." If he refers to The Hospital of December 9, 1911, page 254, he will find it shown that the plan proposed, that is, a pro rata payment by the Health Committees to the hospitals for State cases, plus 10 per cent. for the remuneration of the practitioners engaged, will conserve the voluntary system, whilst it protects the Voluntary Hospitals from abuse and enables them to treat properly those insured persons who need such help.—Ed. The Hospital.

